# Repeatable Self-Healing of a Protective Coating Based on Vegetable-Oil-Loaded Microcapsules

**DOI:** 10.3390/polym14102013

**Published:** 2022-05-15

**Authors:** Young-Kyu Song, Hyun-Woo Kim, Chan-Moon Chung

**Affiliations:** Department of Chemistry, Yonsei University, Wonju 26493, Korea; ssonga0601@naver.com (Y.-K.S.); k.hyunwoo@yonsei.ac.kr (H.-W.K.)

**Keywords:** microcapsule, repeatable self-healing, protective coating, soybean oil, olive oil, viscoelasticity

## Abstract

Generally, microcapsule-based self-healing materials have the limitation of single local self-healing. A few studies have reported repeatable self-healing in these microcapsular materials, but there is a challenge to develop multi-cycle self-healing materials that have the advantages of easier preparation and a more efficient operation. In this work, a mixture of two vegetable oils, soybean and olive oil, was used as a healing agent. The atmospheric oxygen-induced reaction behavior (in the presence of a catalyst) was investigated for various compositions of the vegetable oil mixtures; infrared spectroscopy, recovery testing, and viscoelasticity measurement were performed to find an optimum composition of the healing agent. Microcapsules loaded with soybean oil and catalyst-containing olive oil were separately prepared and used to prepare a dual-capsule self-healing coating. It was demonstrated through optical and scanning electron microscopy that, upon scribing the self-healing coating, the vegetable oils flowed out from microcapsules to self-heal the damaged area. When the healed area of the self-healing coating was re-scribed, self-healing was repeated, which was confirmed by scanning electron microscopy (SEM) and anticorrosion and electrochemical testing. Our new repeatable self-healing coating provides the merits of easy preparation, no need for external intervention such as light irradiation, and an environmentally-friendly nature.

## 1. Introduction

In recent times, great attention has been paid to the self-healing of materials when damage occurs, which extends their functional lifetimes [1,2,3,4,5]. Extensive research in this area has been performed with regard to the enhancement of public safety, reduction of maintenance costs, and environmental protection. Self-healing materials can generally be classified into intrinsic and extrinsic types [1,5]. Intrinsic type self-healing materials can self-heal without external intervention. The intrinsic type has the advantage of repeating self-healing because the chemical or physical healing processes are reversible. In contrast, an extrinsic self-healing material is mostly composed of a matrix material and embedded microcapsules loaded with a healing agent. When extrinsic self-healing materials are damaged, the microcapsules are ruptured, and healing agents flow out and fill the damaged area. Finally, the healing agent solidifies through a chemical reaction, and self-healing is achieved. Compared with the intrinsic system, the microcapsule-type system has the advantage of healing a larger damaged volume [1,6,7]; however, these microcapsule-type systems have the limitation of single local self-healing [1,8,9]; when the healed region is damaged again, repeated self-healing cannot occur because healing agent-loaded microcapsules are depleted and the released healing agent transforms into a hard solid without the self-healing capability. To solve this limitation, vascular type self-healing materials having multiple local healing abilities have been developed [8,10]; however, such systems have disadvantages based on the difficulty in vascular structure fabrication and the need to use an external healing agent supply.

Recently, a few studies have reported repeatable self-healing of microcapsule-type self-healing materials. A cinnamide moiety-containing polydimethylsiloxane healing agent flows out of ruptured microcapsules and photochemically transforms into a viscoelastic substance which has a repeatable self-healing capability [6]. A mixture of 1,1,1-tris(cinnamoyloxymethyl)ethane and 1,1-bis(cinnamoyloxymethyl)ethane compounds was encapsulated to achieve repeatability of light-induced self-healing [11]. An epoxy and a hardener were separately encapsulated within an alginate polymer to prepare a dual-capsule type self-healing material that can provide multiple healing cycles [12]. A hierarchical microcapsule with multi-storage cells was designed to repeatedly release healing agents in the same area [13]; however, these conventional systems are limited to complicated processes for the preparation of the healing agent or microcapsules. In addition, some systems need UV irradiation for (repeatable) self-healing; therefore, there is a challenge to develop microcapsular multicycle self-healing materials which have the merits of easy preparation and efficient operation without UV irradiation.

A vegetable oil, which is a mixture of saturated and unsaturated fatty acids in the form of triglyceride ester, is shown in Figure S1 and Table S1 (see Supplementary Materials). The oil chemical structure generally has 14–22 carbon-containing fatty acid ester chains, each of which consists of 1–3 double bonds of the cis form. Vegetable oils undergo an oxidative crosslinking reaction when exposed to air and are dried (or solidified) through the reaction (see Supplementary Materials). According to the degree of drying, they can be classified into drying oil, semi-drying oil, and non-drying oil. Drying (or semi-drying) oils have been used in industrial applications, such as coatings and paints. In particular, several studies have been reported on the application of drying oils (for example, linseed oil, soybean oil, or tung oil) to microcapsule-type self-healing protective coatings [14,15,16,17,18,19,20,21,22].

Here, a new microcapsule-type repeatable self-healing system based on vegetable-oil healing agents was developed. A drying oil (soybean oil) and non-drying oil (olive oil) were separately encapsulated to prepare a dual-capsule self-healing coating. When the self-healing material is damaged, the vegetable oils would be released and mixed together to produce a viscoelastic substance through the oxidative reaction, leading to the first instance of self-healing. When damage re-occurs in the recovered area, self-healing can be repeated, based on the intrinsic self-healing ability of the viscoelastic substance. That is to say, in this work, the strategy for repeatable self-healing is the combination of extrinsic and intrinsic self-healing. It should be noted that, in the case of the self-healing protective coating, the recovery of the barrier function that protects its substrate from corrosive substances such as water would be more important than the recovery of mechanical strength [23]. Our new self-healing system has several benefits compared with the conventional microcapsule-type repeatable self-healing systems. First, there is no need to synthesize a specialized structure of healing agent or microcapsule, and its preparation does not include complex processes. Second, the system can self-heal through an atmospheric oxygen-induced reaction without light irradiation. Third, the use of natural vegetable oils as the healing agent is eco-friendly.

## 2. Materials and Methods

### 2.1. Materials

Urea, aqueous formaldehyde solution (37 wt%), and resorcinol were used to form microcapsule shell materials and were purchased from Merck-Korea (Seoul, Korea). Poly(ethylene-*alt*-maleic anhydride) (EMA) was used as a surfactant and was purchased from Merck-Korea (Seoul, Korea). Moreover, 1-octanol was used as an anti-forming agent and was purchased from Merck-Korea (Seoul, Korea). Ammonium chloride and sodium hydroxide were used to control pH and were purchased from Duksan Pure Chemicals (Seoul, Korea). Soybean and olive oils were used as core materials (healing agents) and were purchased from CJ Cheiljedang (Seoul, Korea). Cobalt (II) 2-ethylhexanoate (65 wt% solution in mineral spirits) was used as a catalyst and was purchased from Merck-Korea (Seoul, Korea). A white enamel paint (KCl 7200) was purchased from Kunsul Chemical Industrial Co. (Seoul, Korea). All the materials are commercial products and were used without purification.

### 2.2. Instruments

Infrared (IR) spectra were measured with a Fourier transform infrared (FT-IR) spectrophotometer (Spectrum One B, Perkin Elmer Co., Waltham, MA, USA). The viscoelasticity of the vegetable oil reaction products was measured with an Advanced Rheometric Expansion System (ARES, Rheometric Scientific, Piscataway, NJ, USA). The viscoelasticity measurements were conducted at 25 °C with a plate–plate type rheometer in a frequency sweep mode with the frequency oscillating from 0.1 to 1000 rad/s. Microencapsulation was conducted with a NZ-1000 mechanical agitator (Eyela, Tokyo, Japan). Images of the microcapsules and coatings were obtained with a BX-51 microscope (Olympus, Tokyo, Japan). The microcapsule mean diameter was analyzed from 300 data sets using a CC-12 CCD camera (Olympus, Tokyo, Japan) built into the microscope and TS image analysis software (Olympus, Tokyo, Japan). A SU-70 scanning electron microscope (Hitachi, Tokyo, Japan) was used to observe the microcapsules and coating surfaces.

### 2.3. Reaction Behavior Study of Soybean and Olive Oil Mixtures

Vegetable oil mixtures were prepared from soybean oil, olive oil, and a catalyst at various mass ratios (Table S2). The oil mixtures were coated to slide glasses and allowed to react for 6 h under ambient conditions. The reaction behavior of the mixture samples was studied by FT-IR spectroscopy. The conversion degree of the C=C bonds of the mixtures was estimated by an IR band–ratio method [17]. The absorbance of the C–H (in H-C=C-H) stretching band at 3010 cm^−1^ was divided by that of the C=O stretching band at 1746 cm^−1^ which was employed as a reference to normalize the variations. The conversion degree was estimated based on the following Equation (1):

Conversion (%) = [1 − (A_3010_/A_1746_)_after reaction_/(A_3010_/A_1746_)_before reaction_] × 100 (1)

where A_3010_ and A_1746_ are the absorbance values of the 3010 and 1746 cm^−1^ bands, respectively.

### 2.4. Intrinsic Self-Healing Behavior Study of Coated Oil Mixtures

Vegetable oil mixtures were prepared from soybean oil, olive oil, and catalyst at various mass ratios (Table S2). Samples of each vegetable oil mixture were coated on slide glasses and allowed to react for 5 days under ambient conditions. The reacted coatings were scribed with a cutter blade. The recovery time of the scribe was measured for each sample. 

### 2.5. Microencapsulation

A 2.5 wt% aqueous solution of EMA (5 mL) was poured in a 100 mL beaker at 25 °C, and urea (0.504 g), resorcinol (0.050 g), ammonium chloride (0.050 g), and water (20 mL) were added. The mixture’s pH was controlled at 3.5 using 10 wt% NaOH solution. The mixture was stirred at 800, 1000, 1200, 1400, or 1600 rpm, followed by an additional 8 mL of soybean oil or 8 mL of olive oil containing the catalyst solution (1.88 wt%), and an emulsion formed. Surface bubbles were removed by adding a small amount of 1-octanol. After adding the formaldehyde aqueous solution (37%, 1.456 g), the resultant emulsion was heated at 60 °C for 5 h. Vacuum filtration was conducted to isolate the microcapsules. After washing with water and THF, the microcapsules were air dried.

### 2.6. Preparation of Self-Healing and Control Coatings

The enamel paint and the microcapsule mixture (soybean oil-loaded microcapsule:olive oil/catalyst solution-loaded microcapsule = 1.0:0.8 by mass) were mixed together at a ratio of 2:1 by mass. The resultant formulation was coated to one side of individual steel plates or slide glasses, followed by drying for 3 days in an ambient atmosphere. The enamel paint was applied to the other side of the panels. In a similar fashion, a control coating was prepared without adding microcapsules for comparison. The thickness of all the coatings was measured to be ca. 300 μm.

### 2.7. Anticorrosion Test

The steel plate dimensions for the anticorrosion test were 20 × 50 × 0.5 mm^3^. The prepared coatings were cross-scribed using a cutter blade. The damaged coatings were kept for 3 days in an ambient atmosphere to induce self-healing, and then soaked in a sodium chloride (NaCl, 10 wt%) aqueous solution for 72 h to induce the accelerated corrosion process (Figure S3). The coatings were washed with water and dried, and then photographed. Re-scribing was applied to the first scribed and healed areas in the coatings, and the re-damaged coatings were stored for 1 h in an ambient atmosphere. The anticorrosion testing was performed again by soaking the coatings in brine for 72 h with subsequent photography.

### 2.8. Electrochemical Test

The first scribed and healed self-healing and control coatings were prepared as described above. The electrochemical testing was carried out with a μ AUTOLAB TYPE III potentiostat/galvanostat (Metrohm Autolab B.V., Utrecht, The Netherlands) in an electrochemical cell equipped with a platinum counter electrode, a reference electrode (Ag/AgCl in saturated NaCl aqueous solution), and a working electrode (coated steel plate) (Figure S4). After attaching an electrochemical cell to the coating, 1M NaCl aqueous solution was poured. After waiting for 30 min, the steady-state conduction between the coated metal substrate and count electrode at 3 V through the brine was measured. The current passing was recorded with a General Purpose Electrochemical System (GPES) (Metrohm Autolab B.V., Utrecht, The Netherlands). The electrochemical cell was removed, and the healed area was re-scribed. After attaching the electrochemical cell to the coating again, the second electrochemical test was conducted. 

## 3. Results and Discussion

### 3.1. Reaction of Vegetable Oil Mixtures and Properties of Their Reaction Products

In this work, soybean and olive oils were used as healing agents, and a catalyst was added to accelerate the healing reaction, which is described in detail in Supplementary Materials. Soybean and olive oils are classified into drying and non-drying oils, respectively. Soybean oil hardens to a tough solid film through an oxidative crosslinking reaction when exposed to air, but olive oil does not dry and maintains a liquid state. This is due to the fact that the soybean oil molecule has many more C=C bonds, leading to a higher crosslinking density, compared with the olive oil molecule (Table S1 in Supplementary Materials). When soybean and olive oils are mixed at an appropriate ratio, the mixture exhibits desirable reactivity, and its reaction product has suitable viscoelasticity (olive oil was used as a diluent to control the properties of the mixture). In this work, the soybean oil and olive oil/catalyst were separately encapsulated to prepare a dual-capsule self-healing coating system. When damage occurs in this system, each oil flows out of the ruptured capsules and fills the damaged region. The two oils and the included catalyst mix together and undergo oxidative crosslinking, forming a viscoelastic substance. As a result, repeated self-healing can be achieved due to the intrinsic self-healing nature of the viscoelastic material.

**Figure 1:**
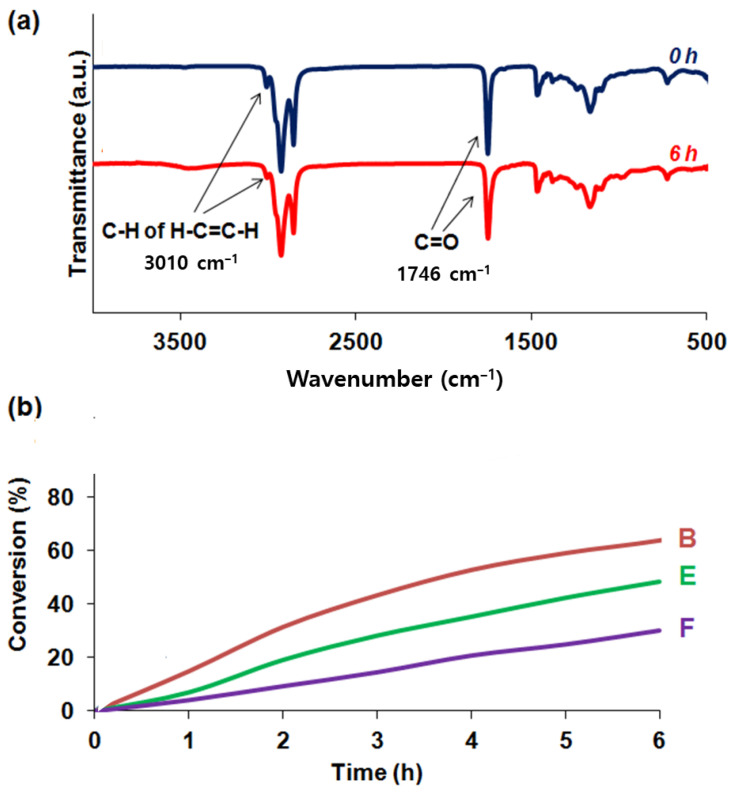
Reaction behavior of the oil mixtures: (**a**) FT-IR spectra of sample E before and after reaction; (**b**) a plot of conversion of C=C bonds vs. reaction time.

The reaction behavior of oil mixtures having various compositions was investigated by FT-IR spectroscopy. After mixing of the two oils and catalyst at various mass ratios (Table S2 in Supplementary Materials), the mixtures were allowed to react under ambient conditions for 6 h. The reaction of the vegetable oil mixtures is shown in Figure 1a. The absorption band at 3010 cm^−1^ corresponds to the C-H (in *cis* H-C=C-H) stretching vibration, which became smaller over time [24]. The absorbance ratios between the 3010 cm^−1^ band of C-H (in *cis* H-C=C-H) and the 1746 cm^−1^ band of C=O, as a reference before and after exposure to air, were compared in order to estimate the conversion degree of the C=C bonds (see Section 2.3). As shown in Figure 1b, the vegetable oil mixtures (samples B, E, and F) showed C=C conversions of 64, 48, and 30%, respectively. The conversion increased with the increasing soybean oil content, which was probably due to the higher C=C bond concentration of soybean oil compared with olive oil. In addition, these results imply that the crosslinking density of the reaction product increases with the increasing soybean oil content.

**Figure 2:**
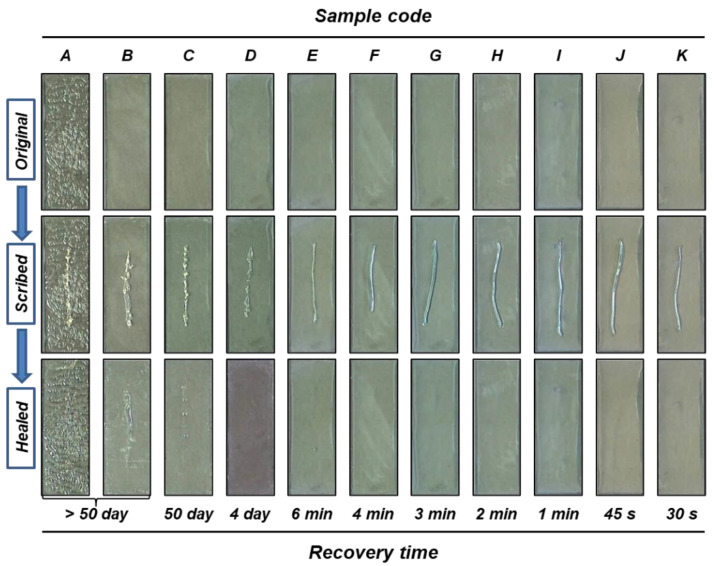
Self-healing behavior of reacted vegetable oil mixture coatings.

To find the optimum composition of the healing agent, the intrinsic self-healing behavior of the reacted vegetable oil mixtures was investigated (Figure 2). The oil mixtures were applied to slide glasses and allowed to react for 5 days under ambient conditions. When the resultant viscoelastic coatings were scribed, recovery of the scribe was observed. The recovery is probably attributable to chain mobility in the viscoelastic reaction products. It is thought that the relative ease of chain mobility in the viscoelastic materials would influence the recovery time [6]. The recovery time increased with the increasing content of soybean oil. This is probably due to the higher crosslinking density of the reaction products which have a higher soybean oil content. Samples A, B, C, and D showed a recovery time of 4 days or longer, which is considered too long. In contrast, samples G, H, I, J, and K showed much higher flowability and a much shorter recovery time. For effective self-healing, the recovery time needs to be short, and the reacted healing agent needs to have relatively low flowability to avoid its permeation through the coating matrix. In this study, sample E offered the advantages of a short recovery time, relatively low flowability, and its composition was considered to be suitable for the purpose of this study. 

**Figure 3:**
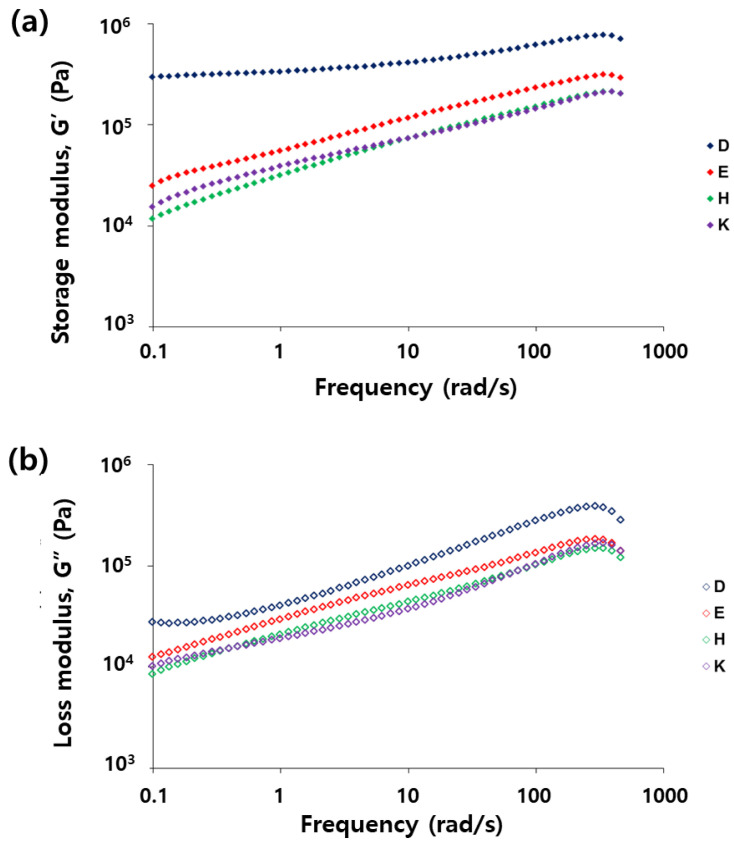
Frequency dependence of (**a**) storage modulus G’ and (**b**) loss modulus G” of the reacted oil mixtures.

An oscillatory shear measurement was performed to investigate the viscoelastic property of the vegetable oil mixtures that had reacted (samples D, E, H, and K) (Figure 3). The storage modulus (G’) and loss modulus (G”) represent the elasticity and viscosity, respectively, and showed an increasing trend as the soybean oil content increased, which is probably due to the higher crosslinking density. As shown in Figure 3a, sample D showed much higher elasticity compared with samples E, H, and K. In addition, the elasticity of sample D was much greater than its viscosity, whereas samples E, H, and K had a similar elasticity and viscosity. The much longer recovery time of sample D compared with those of the other samples (E–K) can be explained using the viscoelastic behavior of the samples.

### 3.2. Microencapsulation

**Figure 4:**
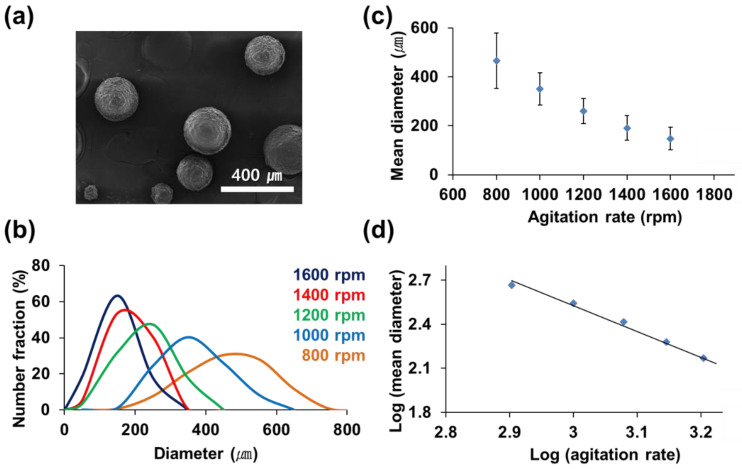
Microencapsulation of soybean oil: (**a**) SEM micrograph of microcapsules prepared at 1200 rpm stirring rate; (**b**) microcapsule size distribution vs. stirring rate; (**c**) average microcapsule diameter vs. stirring rate; (**d**) average microcapsule diameter vs. stirring rate on a log–log scale.

The soybean oil and olive oil/catalyst were separately microencapsulated in oil-in-water emulsions. Urea and formaldehyde were in situ polymerized to form a UF shell wall. During the microencapsulation of soybean oil, the stirring rate was changed from 800 to 1600 rpm while keeping all other conditions constant. The microcapsules’ morphology was observed to be spherical by SEM as shown in Figure 4a. As the stirring rate increased, the size distribution narrowed (Figure 4b), and the average capsule diameter was reduced (Figure 4c,d). The average capsule diameter reduced from 465 to 130 μm as the stirring rate increased from 800 to 1600 rpm. As the stirring rate increased, finer emulsion droplets were obtained, resulting in the narrower size distribution and reduced diameter of microcapsules.

**Figure 5:**
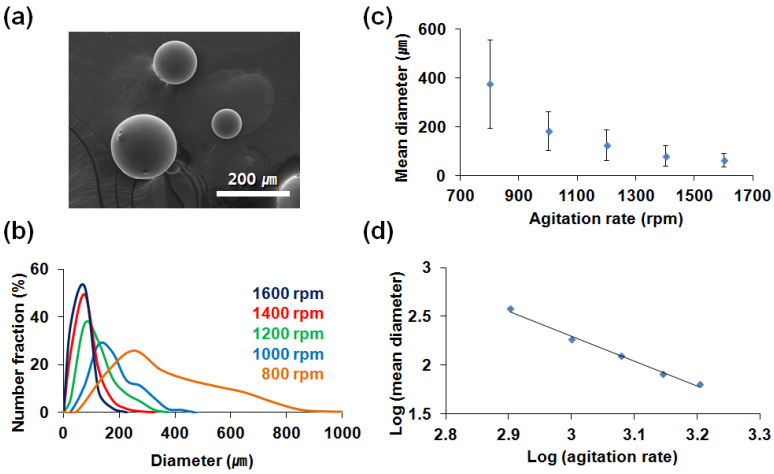
Microencapsulation of olive oil/catalyst: (**a**) SEM micrograph of microcapsules prepared at a 1000 rpm stirring rate; (**b**) microcapsule size distribution vs. stirring rate; (**c**) average microcapsule diameter vs. stirring rate; (**d**) average microcapsule diameter vs. stirring rate on a log–log scale.

During the microencapsulation of the olive oil-containing catalyst, the prepared microcapsules were spherical at all stirring rates from 800 to 1600 rpm (Figure 5a). As the stirring rate increased, the size distribution narrowed (Figure 5b) and the average diameter of the capsules decreased (Figure 5c,d). The average capsule diameter decreased from 376 to 64 μm as the stirring rate increased from 800 to 1600 rpm.

**Figure 6:**
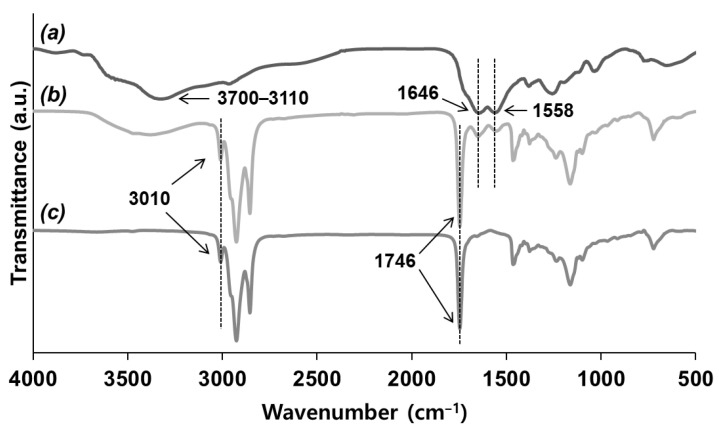
IR spectra of (**a**) UF polymer, (**b**) soybean-loaded microcapsules, and (**c**) soybean oil.

**Figure 7:**
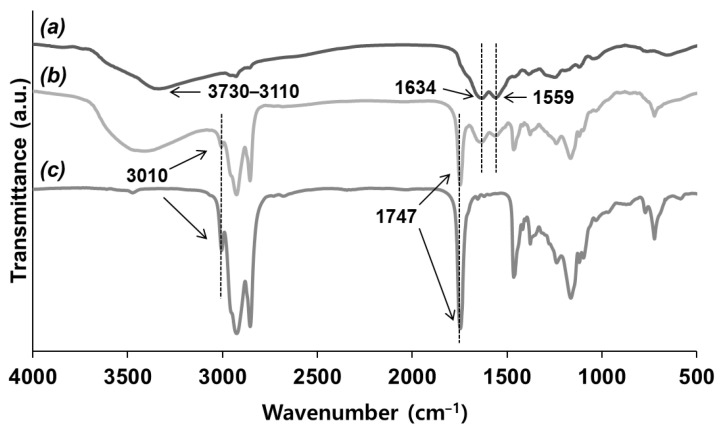
IR spectra of (**a**) UF polymer, (**b**) olive oil/catalyst-loaded microcapsules, and (**c**) olive oil/catalyst solution.

The prepared vegetable oil-loaded microcapsules were characterized by FT-IR spectroscopy. As shown in Figure 6a, a separately prepared UF polymer showed the stretching vibration absorption bands of N-H and O-H groups at 3110~3700 cm^−1^, C=O groups at 1646 cm^−1^, and C-N groups at 1558 cm^−1^. Moreover, soybean oil showed stretching vibration absorption as the C=O groups were at 1746 cm^−1^ and C-H (in cis H-C=C-H) groups were at 3010 cm^−1^ (Figure 6c). The spectrum of the microcapsules showed all these main absorption bands of the UF resin and soybean oil (Figure 6b), suggesting the successful preparation of soybean oil-filled UF microcapsules. The IR spectra of olive oil/catalyst-loaded microcapsules showed a combined absorption of a separately prepared UF resin (3110~3730, 1634, and 1559 cm^−1^) and olive oil (3010 and 1747 cm^−1^), suggesting the formation of olive oil/catalyst-containing microcapsules (Figure 7).

### 3.3. Evaluation of Repeatable Self-Healing Capability

The prepared microcapsules were integrated into a polymer coating to evaluate the repeatable self-healing ability. The soybean oil-loaded and olive oil/catalyst-loaded microcapsules were mixed at a mass ratio of 1:0.8 (this ratio was based on sample E composition). The combined microcapsules were uniformly mixed into a commercial enamel paint formulation at a mass ratio of 1:2. The resultant test formulation was coated to one side of steel panels or slide glasses to obtain a dual-capsule self-healing coating. 

**Figure 8:**
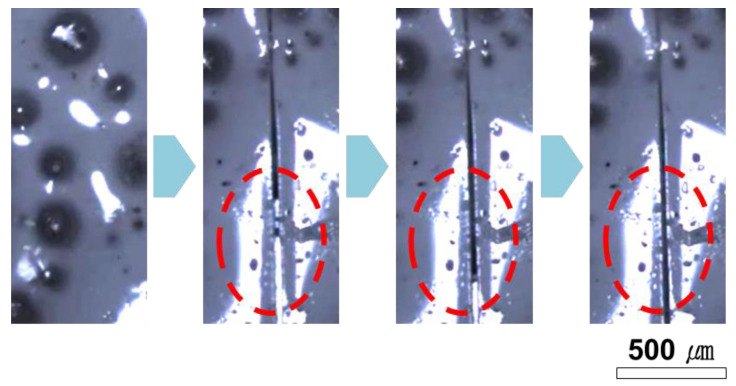
Optical micrographs of a scribed self-healing coating. This shows that the vegetable oils flowed out from ruptured microcapsules and filled the scribe.

For effective self-healing, a healing agent needs to readily flow out from broken microcapsules and fill the damaged area. The release behavior of the healing agent was investigated by optical microscopy as shown in Figure 8. When the dual-capsule self-healing coating was damaged using a cutter blade, the vegetable oils were immediately released and filled the scribe area.

**Figure 9:**
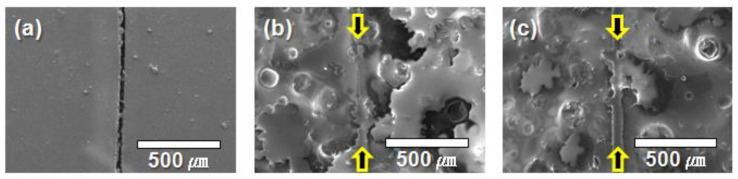
SEM micrographs of the scribed regions in (**a**) a control coating, (**b**) a self-healing coating after the first scribing, and (**c**) a self-healing coating after the second scribing of the healed region.

For the self-healing coating, the repeatability of self-healing was examined by SEM. A steel panel was coated with the coating formulation, and the resulting self-healing coating was scribed and kept under ambient conditions for 3 days to induce a reaction between the released vegetable oils. The SEM image of the scribed area shows that the vegetable oils filled the scribe, indicating that the scribe effectively healed (Figure 9b), whereas the scribe in the control coating remained unfilled (Figure 9a). Next, another self-healing coating was scribed and kept under ambient conditions for 3 days to induce self-healing. When the healed region was scribed again, self-healing repeatedly occurred as shown in Figure 9c. This suggests that intrinsic type self-healing of the viscoelastic healing agent occurred in the re-scribed region.

**Figure 10:**
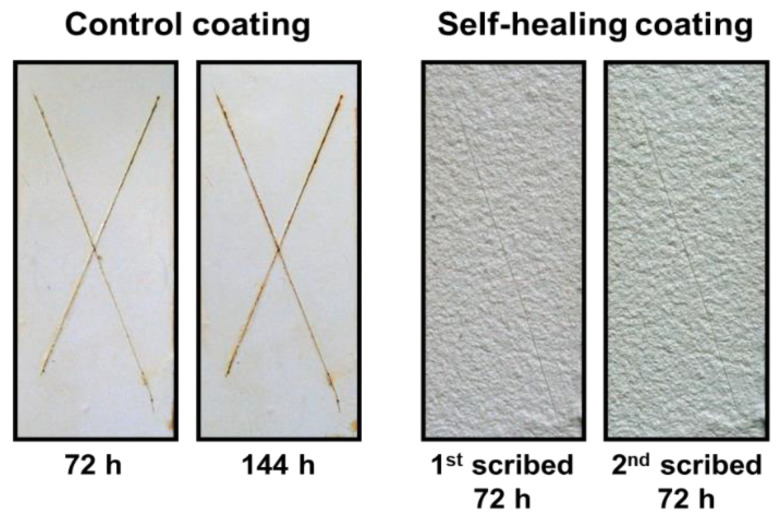
Photos of the coating samples after soaking in a NaCl aqueous solution.

Anticorrosion testing was conducted to further demonstrate the repeatability of the self-healing of the vegetable-oil-based coating (Figure 10). Self-healing coatings on the steel panels were prepared. The coatings were scribed and stored for 3 days in an ambient atmosphere for induction of the reaction of the released vegetable oils. A control coating was treated according to a similar method. The resultant coatings were soaked in a brine for 72 h (Figure S3 in Supplementary Materials). As shown in Figure 10, the control coating corroded, but no visual corrosion was observed in the self-healing coating. This implies that the first scribe in the self-healing coating self-healed, and protection of the steel panel from corrosion was achieved. The first scribed area in the self-healing coating was re-scribed, and the resultant coating specimens were re-soaked in the brine for 72 h. As expected, the control coatings exhibited additional corrosion (total soaking time was 144 h). In contrast, it was confirmed that the self-healing coatings were nearly free of corrosion upon secondary damage. The reacted vegetable oil mixture possesses a viscoelastic property, so repeated self-healing occurred.

**Figure 11:**
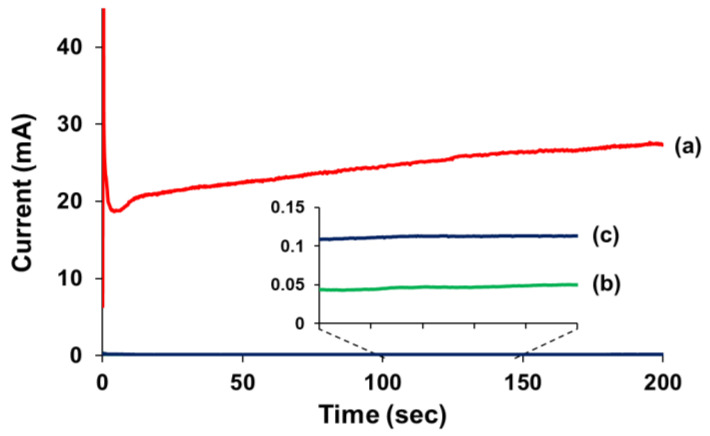
Current vs. time for (**a**) a control coating after scribing, (**b**) a self-healing coating after the first scribing and healing, and (**c**) the self-healing coating after the second scribing of the healed region.

Electrochemical testing provided further definite quantitative evidence of the repeatable self-healing function of the vegetable oil-based coating (Figure 11). Self-healing and control coatings on the steel panels were prepared and scribed. A three-electrode electrochemical cell was fabricated as shown in Figure S4 (see Supplementary Materials). If the scribed coatings self-healed, there would be almost no current passing through the scribed region. The scribed control coating showed a current flow of 24 mA (Figure 11a), indicating that a high current flowed through the unfilled scribe hole. In contrast, the self-healing coating sample exhibited a very low current flow of 0.04 mA after the first scribing and of 0.11 mA after the second scribing (Figure 11b,c, respectively). These results indicate that the first and second scribes in the self-healing coating successfully healed.

## 4. Conclusions

Two vegetable oils, soybean oil as a drying oil and olive oil as a non-drying oil, were used as healing agents. The atmospheric oxygen-induced reaction behavior (in the presence of a catalyst) of the various composition vegetable oil mixtures was studied by FT-IR spectroscopy, and the properties of the reaction products were investigated by recovery testing and viscoelasticity measurement. As the soybean oil concentration increased, the mixtures showed a higher C=C conversion (i.e., higher crosslinking density). When scribed, the reaction product coatings showed an intrinsic self-healing behavior due to their viscoelastic properties. In addition, the reaction products with a higher soybean oil concentration showed a longer recovery time and higher G’ and G”. It was considered that sample E had the most suitable composition as a healing agent based on its short recovery time and relatively low flowability. The separately prepared soybean oil and catalyst-containing olive oil microcapsules were used for the preparation of a dual-capsule self-healing coating. Optical microscopy and SEM studies indicated that, when the self-healing coating was scribed, the vegetable oils flowed out from microcapsules and healed the scribed region. Repeatable self-healing was successfully demonstrated by SEM, anticorrosion testing, and electrochemical testing. Our new self-healing system offers the advantages of simple preparation, atmospheric oxygen-induced healing without UV irradiation, and an environmentally-friendly nature.

## Data Availability

Not applicable.

## Supplementary Materials

The following supporting information can be downloaded at: https://www.mdpi.com/article/10.3390/polym14102013/s1, Figure S1: A triglyceride chain containing three fatty acid chains joined by a glycerol center; Figure S2: (A) Schematic illustration of the crosslinking process of linoleic chain. (B) Electronic state of cobalt in cobalt(II) 2-ethylhexanoate; Figure S3: Schematic illustration of anticorrosion test; Figure S4: Schematic illustration of electrochemical test; Table S1: Main fatty acid contents in different vegetable oils; Table S2: Mass ratios of vegetable oil mixtures used in the reaction behavior study.
