# Supplementary material for: Repeatable Self-Healing of a Protective Coating Based on Vegetable-Oil-Loaded Microcapsules
Source: Polymers (Basel). 2022 May 15;14(10):2013. doi: 10.3390/polym14102013 (PMC9146027; doi:10.3390/polym14102013)
Supplement: Supplementary file 1 [file polymers-14-02013-s001.zip › polymers-1709819-supplementary.pdf]

Supplementary Materials: Repeatable Self-Healing of a Protective Coating Based on Vegetable-Oil-Loaded Microcapsules

Young-Kyu Song, Hyun-Woo Kim and Chan-Moon Chung*

1. Vegetable oils and preparation of their mixtures

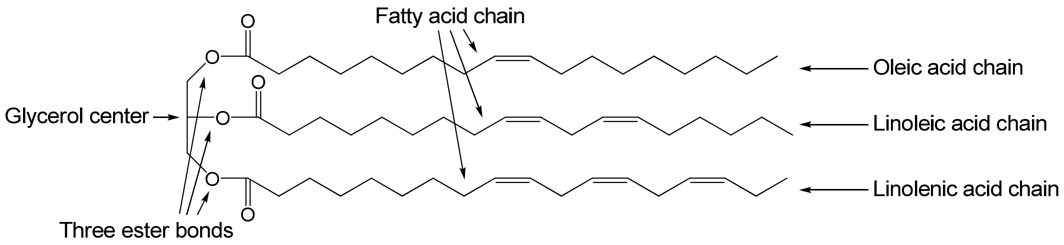

Figure S1. A triglyceride chain containing three fatty acid chains joined by a glycerol center.

Table S1. Main Fatty Acid Contents in Different Vegetable Oils.

Fatty acid	[#C:#DB*]	Linseed oil	Soybean oil	Olive oil
Palmitic	16:0	5.5	11.0	13.7
Stearic	18:0	3.5	4.0	2.5
Oleic	18:1	19.1	23.4	71.1
Linoleic	18:2	15.3	53.3	10.0
Linolenic	18:3	56.6	7.8	0.6
Average #DB/triglyceride	-	6.6	4.6	2.8

*#C stands for number of carbon atoms in chain and #double bond (DB) stands for the number of double bonds in that chain.

Table S2. Mass ratios of vegetable oil mixtures used in the reaction behavior study.

Sample Code	Soybean Oil (g)	Catalyst solution (g) [a]	Olive Oil (g)
A	0.9846	0.0154	0.0000
B	0.9846	0.0154	0.2000
C	0.9846	0.0154	0.4000
D	0.9846	0.0154	0.6000
E	0.9846	0.0154	0.8000
F	0.9846	0.0154	1.0000
G	0.9846	0.0154	1.2000
H	0.9846	0.0154	1.4000
I	0.9846	0.0154	1.6000
J	0.9846	0.0154	1.8000
K	0.9846	0.0154	2.0000

[a] 65 wt% solution of cobalt (II) 2-ethylhexanoate in mineral spirits.

2. Oxidative crosslinking reaction of vegetable oils in the presence of a cobalt catalyst

It is considered that the oxidative crosslinking reaction proceeds in the presence of the cobalt catalyst as shown in Figure S2A (refer to D. Li et al., Prog. Org. Coat. 2022, 166, 106776). In the oxidation process of linoleic chain with O_2, the valence of cobalt ion changes between trivalent and divalent (Figure S2B). Both are not stable in a low-energy state: they are neither half-filled nor fully filled.

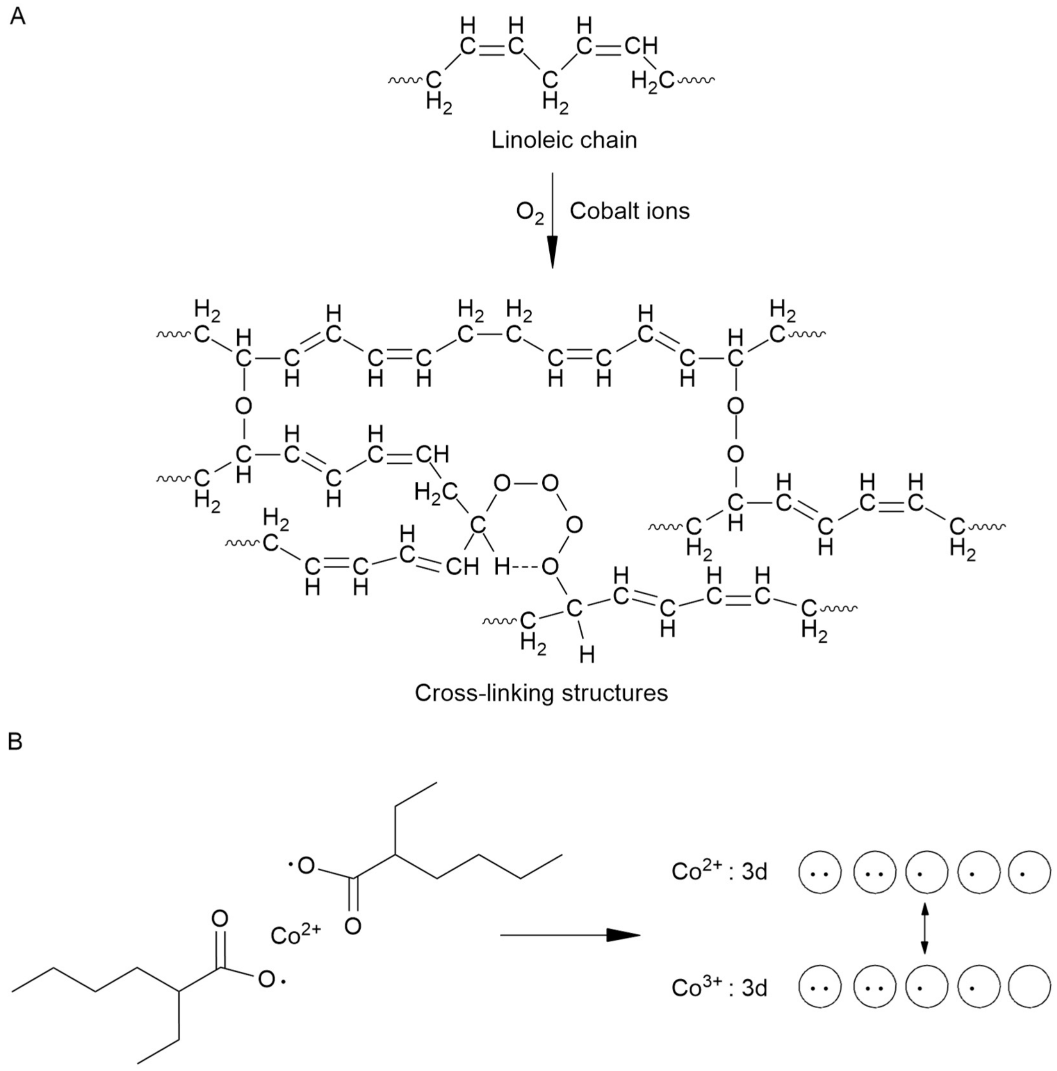

Figure S2. (A) Schematic illustration of the crosslinking process of linoleic chain. (B) Electronic state of cobalt in cobalt(II) 2-ethylhexanoate.

Catalytic process of cobalt ion is shown below(chemcal equations (1)~(4)).

$$RH + Co^{3+} \longrightarrow R\cdot + Co^{2+} + H^{+} \quad (1)$$

$$R\cdot + O_2 + RH \longrightarrow ROOH + R\cdot \quad (2)$$

$$ROOH + Co^{2+} \longrightarrow Co^{3+} + RO\cdot + OH^{-} \quad (3)$$

$$ROOH + Co^{3+} \longrightarrow Co^{2+} + ROO\cdot + H^{+} \quad (4)$$

Co^{2+} is oxidized to Co^{3+} in the air, and redox reaction occurs with the unsaturated fatty acid groups in soybean and olive oils, accelerating the generation and decomposition of peroxides into free radicals. The generated free radicals (R•, RO• and ROO•) are further coupled into crosslinked macromolecules.

3. Evaluation of repeatable self-healing performance

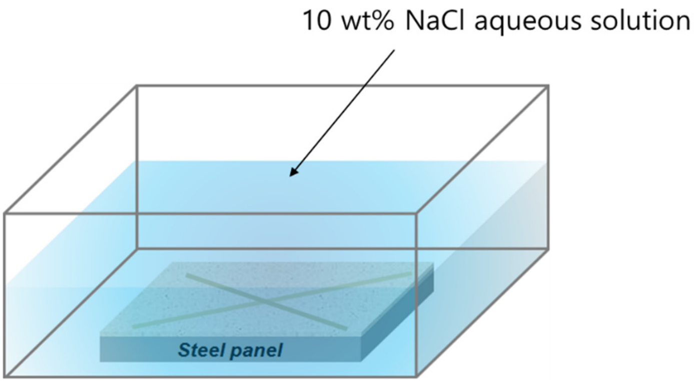

Figure S3. Schematic illustration of anticorrosion test.

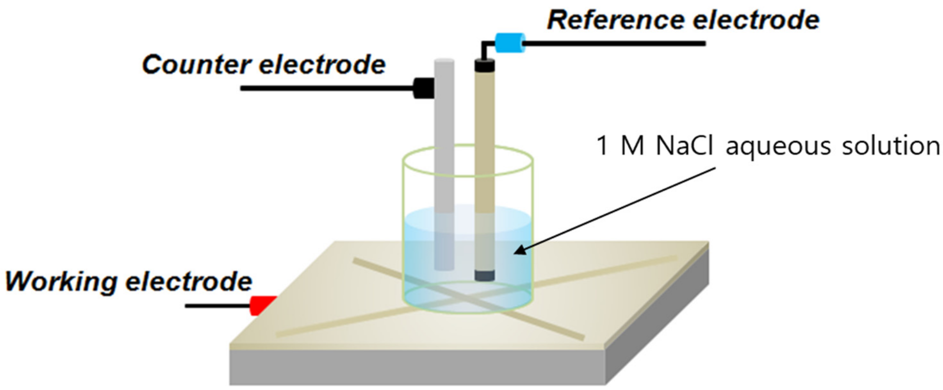

Figure S4. Schematic illustration of electrochemical test.
